# Altered Follicular Fluid Metabolic Pattern Correlates with Female Infertility and Outcome Measures of In Vitro Fertilization

**DOI:** 10.3390/ijms22168735

**Published:** 2021-08-14

**Authors:** Giacomo Lazzarino, Romina Pallisco, Gabriele Bilotta, Ilaria Listorti, Renata Mangione, Miriam Wissam Saab, Giuseppe Caruso, Angela Maria Amorini, Maria Violetta Brundo, Giuseppe Lazzarino, Barbara Tavazzi, Pasquale Bilotta

**Affiliations:** 1 UniCamillus—Saint Camillus International University of Health Sciences, Via di Sant’Alessandro 8, 00131 Rome, Italy; giacomo.lazzarino@unicamillus.org; 2 Alma Res Fertility Center, Laboratory of Andrology and Embriology, Via Parenzo 12, 00198 Rome, Italy; laboratorio@almares.it (R.P.); bilotta.oblomov@gmail.com (G.B.); ilaria.listorti@gmail.com (I.L.); 3 Department of Basic Biotechnological Sciences, Intensive and Perioperative Clinics, Catholic University of Rome, Largo F. Vito 1, 00168 Rome, Italy; renata.mangione@unicatt.it; 4 Fondazione Policlinico Universitario A. Gemelli IRCCS, Largo A. Gemelli 8, 00168 Rome, Italy; 5 Department of Biomedical and Biotechnological Sciences, Division of Medical Biochemistry, University of Catania, Viale A. Doria 6, 95125 Catania, Italy; mirisaab@gmail.com (M.W.S.); amorini@unict.it (A.M.A.); 6 Department of Drug and Health Sciences, University of Catania, Viale A. Doria 6, 95125 Catania, Italy; forgiuseppecaruso@gmail.com; 7 Department of Biology, Geology and Environmental Sciences, Section of Animal Biology, University of Catania, Via Androne 81, 95124 Catania, Italy; mvbrundo@unict.it; 8 Department of Biomedical and Biotechnological Sciences, LTA-Biotech srl, Viale Don Orione 3D, 95047 Paternò, Italy; 9 Alma Res Fertility Center, Obstetrics and Gynecology, Via Parenzo 12, 00198 Rome, Italy; pasquale.bilotta@almares.it

**Keywords:** amino acids, antioxidants, assisted reproduction techniques, biomarkers, energy metabolites, female infertility, follicular fluid, oxidative/nitrosative stress, targeted metabolomics

## Abstract

Nearly 40–50% of infertility problems are estimated to be of female origin. Previous studies dedicated to the analysis of metabolites in follicular fluid (FF) produced contrasting results, although some valuable indexes capable to discriminate control groups (CTRL) from infertile females (IF) and correlate with outcome measures of assisted reproduction techniques were in some instances found. In this study, we analyzed in blind FF of 35 control subjects (CTRL = patients in which inability to obtain pregnancy was exclusively due to a male factor) and 145 IF (affected by: endometriosis, *n* = 19; polycystic ovary syndrome, *n* = 14; age-related reduced ovarian reserve, *n* = 58; reduced ovarian reserve, *n* = 29; unexplained infertility, *n* = 14; genetic infertility, *n* = 11) to determine concentrations of 55 water- and fat-soluble low molecular weight compounds (antioxidants, oxidative/nitrosative stress-related compounds, purines, pyrimidines, energy-related metabolites, and amino acids). Results evidenced that 27/55 of them had significantly different values in IF with respect to those measured in CTRL. The metabolic pattern of these potential biomarkers of infertility was cumulated (in both CTRL and IF) into a Biomarker Score index (incorporating the metabolic anomalies of FF), that fully discriminated CTRL (mean Biomarker Score value = 4.00 ± 2.30) from IF (mean Biomarker Score value = 14.88 ± 3.09, *p* < 0.001). The Biomarker Score values were significantly higher than those of CTRL in each of the six subgroups of IF. Posterior probability curves and ROC curve indicated that values of the Biomarker Score clustered CTRL and IF into two distinct groups, based on the individual FF metabolic profile. Furthermore, Biomarker Score values correlated with outcome measures of ovarian stimulation, in vitro fertilization, number and quality of blastocysts, clinical pregnancy, and healthy offspring. These results strongly suggest that the biochemical quality of FF deeply influences not only the effectiveness of IVF procedures but also the following embryonic development up to healthy newborns. The targeted metabolomic analysis of FF (using empowered Redox Energy Test) and the subsequent calculation of the Biomarker Score evidenced a set of 27 low molecular weight infertility biomarkers potentially useful in the laboratory managing of female infertility and to predict the success of assisted reproduction techniques.

## 1. Introduction

Infertility is defined as the failure to achieve a clinical pregnancy after at least 12 months of regular unprotected sexual intercourse. Nearly 40–50% of infertility problems are estimated to be of female origin, and approximately 30% of cases are of male origin. A further 20–25% of couples suffer from unexplained infertility [1]. The most common causes of female infertility are endometriosis (EM), polycystic ovary syndrome (PCOS), age-dependent and age-independent reduced ovarian reserve (ROR) [2].

When infertile females undergo in vitro fertilization (IVF), it is known that only 32% of IVF cycles result in pregnancy [2] and that only a fraction of the oocytes retrieved in an IVF cycle (7%) has the potential to develop into a viable embryo destined for a live birth [3]. Therefore, improving the quality of oocytes and the selection methods for fertilization would aid in overcoming the ethical, legal, and storage implications of the current overproduction of embryos. In recent years, several studies have been dedicated to carrying out accurate, non-invasive, and cost-effective predictive tests of oocyte or embryo developmental potential to increase pregnancy rates while employing elective single-embryo transfer. In this light, increasing interest has been developed in biochemical analyses dedicated to exploring the metabolic composition of follicular fluid (FF) that may deeply affect both oocytes and embryo quality [4].

FF is the biological fluid that supports the maturation of the oocyte, influences the effectiveness of oocyte fertilization, and affects embryo development of fertilized oocytes. FF is easily accessible for withdrawals during oocyte pick-up, is abundant, and, above all, it is currently considered a waste product. FF may represent an optimal biological sample to perform non-invasive biochemical analyses aimed to predict oocyte quality and, potentially, even that of an embryo.

FF is a mixture of secretions from granulosa and theca cells and compounds diffusing across the basement membrane from plasma, including proteins, hormones, metabolites, and toxins [4]. Granulosa cells play an active role in the overall composition of FF by selectively filtering and perhaps transforming metabolites [5,6]. On the other hand, it has also been observed that alterations in FF composition may be due to systemic metabolic changes detectable by measuring changes of specific serum analytes [7]. The limited permeability and shape of the follicle walls restrict the entry of many plasma proteins so that their levels are only one-third of those in plasma [4]. FF is probably involved in several physiological processes, providing supplies of nourishment to the oocyte and the granulosa cells [5], and offering some protection during both oocyte development and ovulation. FF composition is certainly connected to the biochemical activity of the follicle; therefore, the evaluation of compounds of biochemical relevance may provide useful information about the growth and differentiation of the follicle itself [8]. It is therefore highly possible that qualitative and quantitative alterations in FF composition may affect the quality of the oocyte and the success of IVF procedures.

Due to the multiplicity of their biochemical functions, the determination of low molecular weight compounds in FF appears of particular relevance to highlight the quality of this biological fluid. This category of compounds includes substances directly (glucose) or indirectly (some amino acids) involved in catabolic reactions connected to energy production, catabolites of cell metabolism reflecting cell bioenergetic (lactate, purines, and pyrimidines), water- and fat-soluble antioxidants involved in defense mechanisms towards oxidative/nitrosative stress, as well as waste products of potential toxicity to the oocyte (ammonia, lactate).

In recent years, several studies have been carried out to determine the metabolic FF profile of women with infertility due to different causes. Using an untargeted metabolomic approach, with the aid of high-resolution proton nuclear magnetic resonance (^1^H-NMR) [9,10] or liquid/gas chromatography-mass spectrometry [5,11], it has been shown that alteration of some metabolites in the FF of infertile females (IF) were affected by PCOS [12], ROR [13] and EM [14,15]. Some metabolomic studies have even found significant differences in IF with negative IVF outcomes compared to IF with positive IVF outcomes [16,17,18]. Although the aforementioned studies are frequently characterized either by a limited number of patients, or by results referring to alterations involving secondary metabolism, or by the lack of outcome measures of biological parameters of IVF and clinical pregnancy, their results reinforced the concept that measuring metabolites in FF may be crucial to highlight deficiencies or excess of specific compounds in infertile females, that may help to explain the high rate of failure of IVF and to develop potentially effective pharmacological treatments.

In a multicenter study using a targeted metabolomic approach, we have previously shown that the concentrations of various low-molecular-weight compounds in seminal plasma of infertile males (ascorbic acid, *all-trans* retinoic acid, *all-trans* retinol, α-tocopherol, γ-tocopherol, total carotenoids, malondialdehyde, 8-hydroxy-2′–deoxyguanosine, nitrites, nitrates, creatinine, cytosine, cytidine, uracil, β-pseudouridine, hypoxanthine, xanthine, uridine, inosine, guanine, and guanosine) are different from those recorded in control fertile males [19]. Furthermore, cumulating the differences in metabolite concentrations into a Biomarker Score, we also showed that these biochemical alterations allowed to discriminate males with idiopathic infertility from those with anomalies in the spermiogram [19].

On the basis of the aforementioned, we undertook the present targeted metabolomic study to determine whether the FF of fertile and infertile females had different profiles of specific metabolites, included in the categories of water- and fat-soluble antioxidants, of free amino acids, of compounds representative of oxidative/nitrosative stress, of a nutrient substrate (glucose), of waste products (lactate and ammonia), and of catabolites related to energy metabolism (purines, pyrimidines, creatinine). Additional aims of the study were to determine whether females with different clinical infertilities had different biochemical signatures of the aforementioned compounds in their FF and to assess whether biochemical differences in FF of infertile females correlated with the main outcome measures useful to assess successful IVF.

## 2. Results

### 2.1. Clinical Features of Enrolled Subjects

The main clinical features of the FF donors, as well as the administration protocols for ovarian stimulation, are reported in Table 1.

No age differences were observed by comparing the group of CTRL with that of pooled IF, whilst the subgroup of AR-ROR, as expected, was composed of patients significantly older than both CTRL and any other subgroup of IF (*p* < 0.01). Significantly lower basal FSH serum levels were found in the subgroups of EM and PCOS. Pooled IF had significantly lower values of both the number of retrieved and mature oocytes. After categorization into the different clinical subgroups, PCOS only had values of both parameters equal to those measured in the group of CTRL.

### 2.2. Concentrations of Metabolites in FF of CTRL and IF

The mean concentrations of the compounds under evaluation in FF of CTRL are reported in Table 2 and Table 3.

Using the values found in the FF of the subjects of the CTRL group, it was possible to calculate the 10–90% percentiles for each metabolite, therefore determining their physiologic concentration ranges under healthy fertility conditions and useful to perform comparisons with values obtained in FF samples of IF. When performing the statistical comparison of the values of the 55 compounds in FF samples of the two groups, it was found that the concentrations of 27 of these metabolites in FF of CTRL were significantly different from those measured in FF of pooled IF (Table 4).

Figure S1 in Supplementary Materials (panels A–N) shows the box plots (reporting, minimum, maximum, median, first quartile, third quartile, and significance) of the compounds related to energy metabolism (glucose, lactate), purines (hypoxanthine, xanthine), pyrimidines (uracil, β-pseudouridine, cytosine, and cytidine), antioxidant defenses (ascorbate and GSH), and oxidative/nitrosative stress (MDA, 8-OH-dG, nitrite and nitrate) that had significantly different concentrations in FF of CTRL from those found in FF of IF (*p* < 0.001).

Similarly, Figure S2 in Supplementary Materials (panels A–M) shows the box plots of fat-soluble vitamins (A, D, and E) and antioxidants (CoQ_10_ and total carotenoids), as well as of amino acids (Ser, Thr, Arg, Val, Met, Trp, Ile and Leu) having significantly different values in FF samples of the two groups of CTRL and IF (*p* < 0.001).

**Figure 1:**
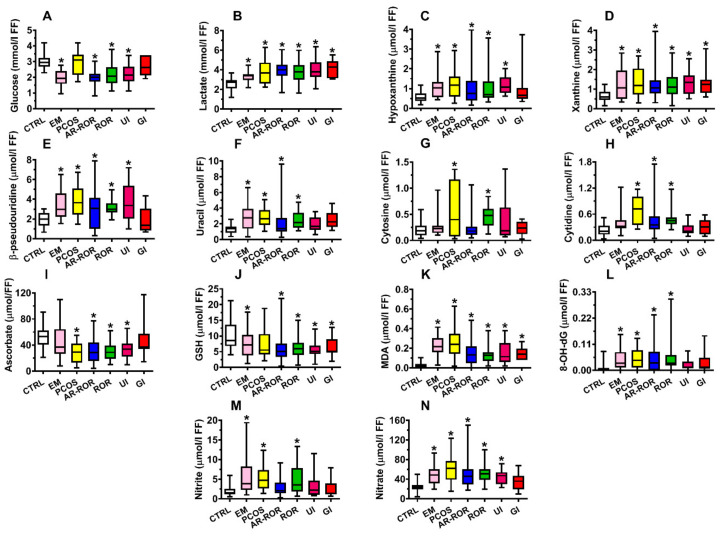
Box plots reporting median, 1st and 3rd quartiles, minimum and maximum values of the concentrations of glucose (**A**), lactate (**B**), hypoxanthine (**C**), xanthine (**D**), β-pseudouridine (**E**), uracil (**F**), cytosine (**G**), cytidine (**H**), ascorbate (**I**), GSH (**J**), MDA (**K**), 8-OH-dG (**L**), nitrite (**M**), and nitrate (**N**) detected in FF of 35 control subjects (CTRL) and in infertile females categorized into endometriosis (EM, *n* = 19), polycystic ovary syndrome (PCOS, *n* = 14), age-related reduced ovarian reserve (AR-ROR, *n* = 58), reduced ovarian reserve (ROR, *n* = 29), unexplained infertile (UI, *n* = 14) and genetic infertile (GI, *n* = 11), according to their clinical diagnosis. Energy-related metabolites (**A**,**B**); purines (**C**,**D**); pyrimidines (**E**–**H**); water-soluble antioxidants (**I**,**J**); oxidative stress indexes (**K**,**L**); nitrosative stress indexes (**M**,**N**). * Significantly different from CTRL, *q* < 0.005.

**Figure 2:**
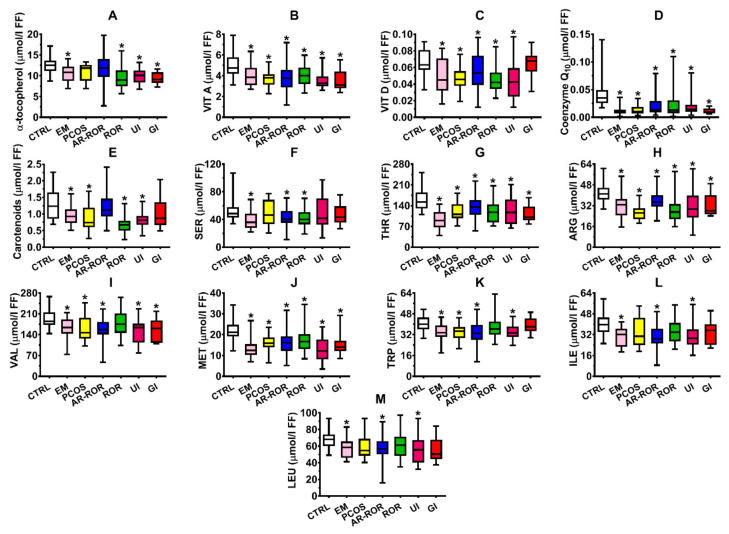
Box plots reporting median, 1st and 3rd quartiles, minimum and maximum values of the concentrations of α-tocopherol (**A**), vitamin A (**B**), vitamin D (**C**), coenzyme Q_10_ (**D**), carotenoids (**E**), serine (**F)**, threonine (**G**), arginine (**H**), valine (**I**), methionine (**J**), tryptophan (**K**), isoleucine (**L**), and leucine (**M**) detected in FF of 35 control subjects (CTRL) and in infertile females categorized into endometriosis (EM, *n* = 19), polycystic ovary syndrome (PCOS, *n* = 14), age-related reduced ovarian reserve (AR-ROR, *n* = 58), reduced ovarian reserve (ROR, *n* = 29), unexplained infertile (UI, *n* = 14) and genetic infertile (GI, *n* = 11), according to their clinical diagnosis. Fat-soluble vitamins and antioxidants (**A**–**E**); amino acids (**F**–**M**). * Significantly different from CTRL, *q* < 0.005.

When the group of IF was categorized according to the differential diagnosis of infertility, allowing to obtain 6 subgroups of IF (EM, PCOS, AR-ROR, ROR, UI, and GI), it was possible to observe that the various subgroups had specific patterns of alterations in the values of the aforementioned compounds in their FF (Figure 1, panels A–N and Figure 2, panels A–M).

### 2.3. The Biomarker Score in CTRL and IF

**Figure 3:**
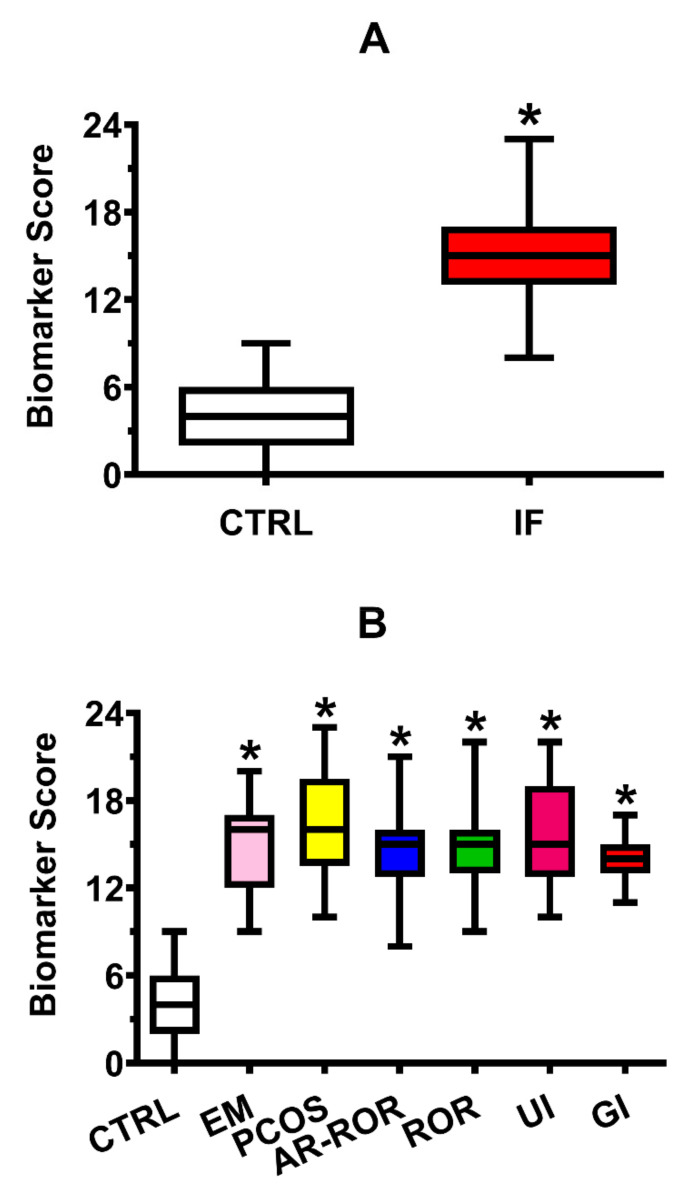
Box plots reporting the Biomarker Score values calculated in: (**A**) the group of 35 control subjects (CTRL) and the group of 145 infertile females (IF) pooled into a single group of infertility, independently on their clinical diagnosis; (**B**) 35 control subjects (CTRL) and infertile females categorized into endometriosis (EM, *n* = 19), polycystic ovary syndrome (PCOS, *n* = 14), age-related reduced ovarian reserve (AR-ROR, *n* = 58), reduced ovarian reserve (ROR, *n* = 29), unexplained infertile (UI, *n* = 14) and genetic infertile (GI, *n* = 11), according to their clinical diagnosis. * In (**A**), significantly different from CTRL, *p* < 0.001. * In (**B**), significantly different from CTRL, *q* < 0.005.

Using the values of the 10–90% percentiles reported in Table 2 and Table 3, concentrations of each of the 27 metabolites (having the meaning of biomarkers of female infertility) of each subject of both CTRL and IF groups scored 0 (if falling within the 10–90% percentiles range of controls) or 1 (if falling outside the 10–90% percentiles range of controls). Therefore, for each subject of the two groups, a Biomarker Score, cumulating the alterations of the 27 metabolites in FF samples, was calculated as the sum of the different metabolite scores. In the group of CTRL, the mean ± S.D. of the Biomarker Score was 4.00 ± 2.30, whilst that in the group of IF was 14.88 ± 3.09 (*p* < 0.001). The box plot of the Biomarker Score values determined in the two groups is illustrated in Figure 3 (Panel A). In the same Figure 3 (Panel B), it is also shown the box plot for the Biomarker Score calculated in CTRL and in IF categorized into the six subgroups, according to the respective clinical diagnosis of infertility.

**Figure 4:**
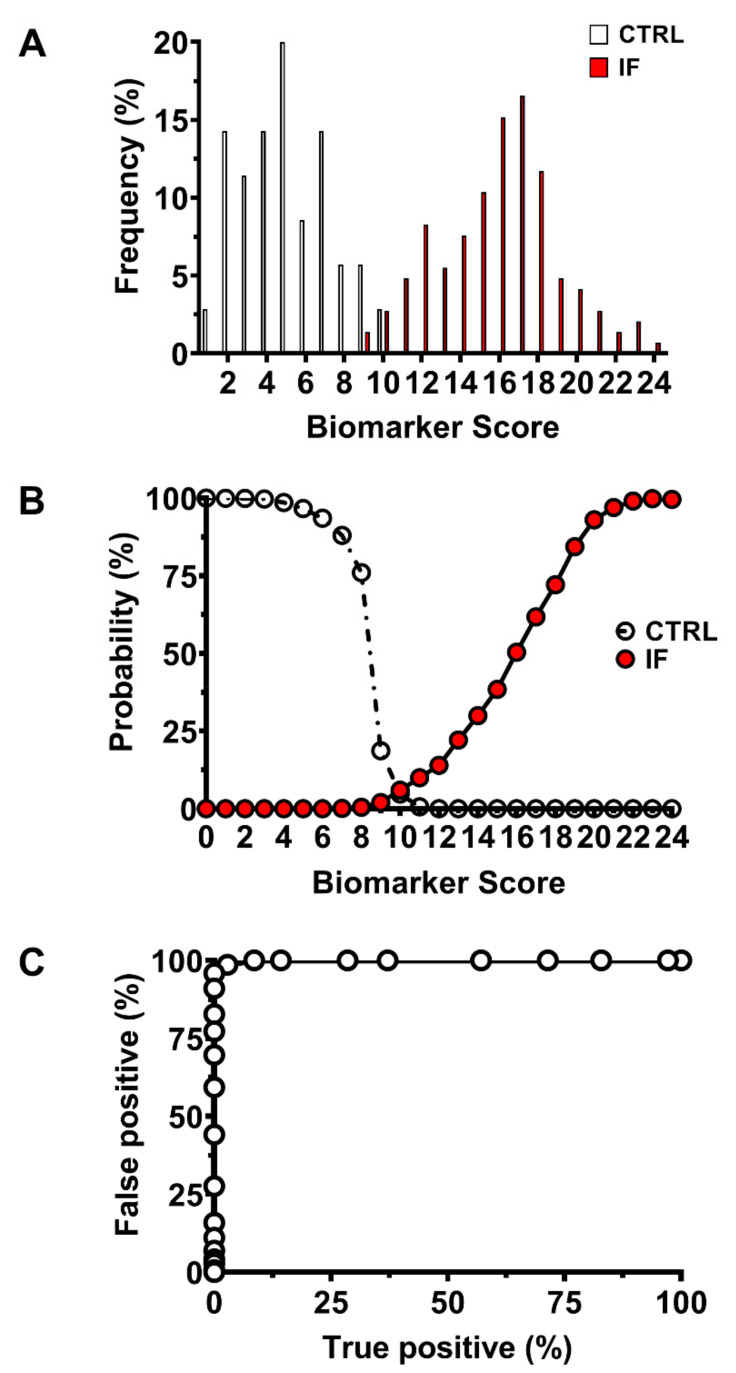
Distribution frequency (**A**), posterior probability curves (**B**), and the ROC curve of the Biomarker Score values (**C**) calculated in the group of 35 control subjects (CTRL) and in the group of 145 infertile females (IF) pooled into a single group of infertility, independently on their clinical diagnosis.

As shown in Figure 4 (Panel A), when plotting the distribution frequency of the Biomarker Score values in the groups of CTRL and IF, it was found that nearly 97.5% of CTRL had Biomarker Score ranging from 0 to 9, whilst the Biomarker Score of the 97.5% of IF ranged from 11 to 23, strongly indicating that values of this index cumulating the biochemical anomalies in FF are able to cluster CTRL (in which infertility was due to a male factor only, and that may high presumably be considered as fertile FF donors) and infertile females into two distinguishable populations. This evidence is corroborated by the data illustrated in Figure 4 (Panel B) and obtained by calculating the posterior probability curves for each patient to be assigned to CTRL or IF, according to the Bayes theorem [20].

It can easily be observed that any patient scoring a Biomarker Score value ≤ 9 belongs to the group of CTRL. Conversely, patients scoring a Biomarker value ≥ 11 can certainly be assigned to the group of IF. Further confirmation of the power of the Biomarker Score as a tool to discriminate the biochemical characteristics of FF is represented by the ROC curve shown in Figure 4 (Panel C), indicating sensitivity close to 100%, probability of false-positive almost near to 0% and specificity also close to 100%. Raw data of the Biomarker Score and details to calculate the ROC curve of Figure 4 (Panel C) are given in Supplementary Materials in Tables S1–S3.

### 2.4. The Biomarker Score of FF Correlates with Biological and Clinical Parameters of IVF and Pregnancy

**Figure 5:**
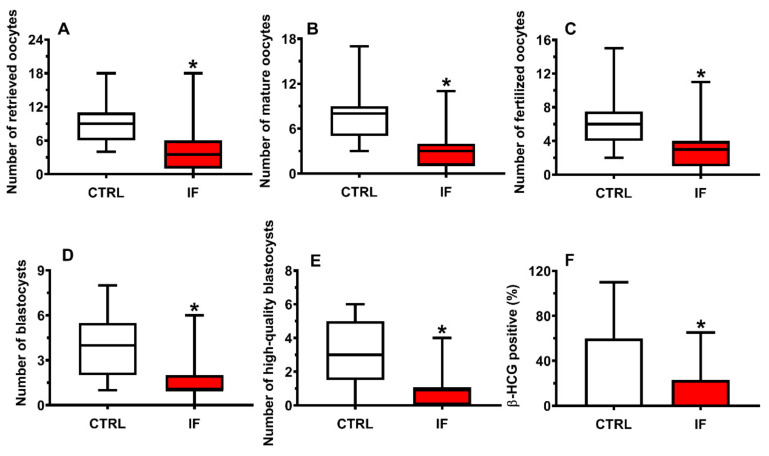
Box plots reporting the median, 1st and 3rd quartiles, minimum and maximum values of the number of retrieved oocytes (**A**), number of mature oocytes (**B**), number of fertilized oocytes (**C**), number of blastocysts (**D**), number of high-quality blastocysts (**E**), and % of β-HCG positive patients (**F**) in 35 control subjects (CTRL) and 145 infertile females (IF) pooled into a single group of infertility, independently of their clinical diagnosis. * Significantly different from CTRL, *p* < 0.001.

The number of retrieved oocytes, mature oocytes, fertilized oocytes, blastocysts, high-quality blastocysts, and β-HCG positive in CTRL and IF patients are shown in the box plots of Figure 5 (Panels A–F). The group of IF patients had significantly lower values of any of these parameters when compared to those found in CTRL (*p* < 0.001).

**Figure 6:**
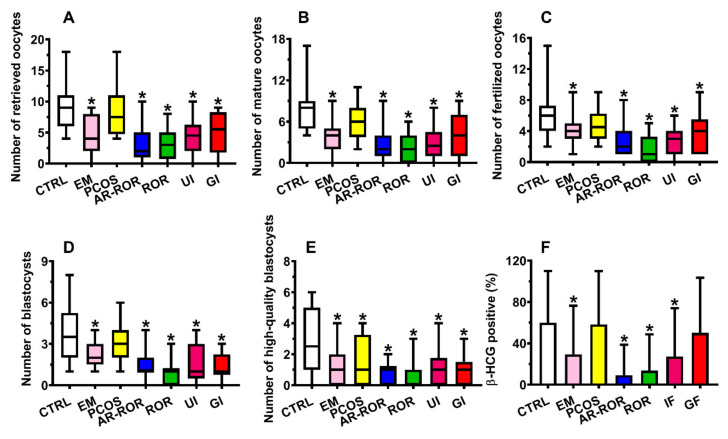
Box plots reporting the median, 1st and 3rd quartiles, minimum and maximum values of the number of retrieved oocytes (**A**), number of mature oocytes (**B**), number of fertilized oocytes (**C**), number of blastocysts (**D**), number of high-quality blastocysts (**E**), and % of β-HCG positive patients (**F**) in 35 control subjects (CTRL) and in infertile females categorized into endometriosis (EM, *n* = 19), polycystic ovary syndrome (PCOS, *n* = 14), age-related reduced ovarian reserve (AR-ROR, *n* = 58), reduced ovarian reserve (ROR, *n* = 29), unexplained infertile (UI, *n* = 14) and genetic infertile (GI, *n* = 11), according to their clinical diagnosis. * Significantly different from CTRL, *q* < 0.001.

By categorizing IF patients into the 6 groups of infertility (Figure 6, Panels A–F), all groups, but that of PCOS, had still statistically different values of the number of retrieved oocytes, mature oocytes, fertilized oocytes, and blastocysts from those of CTRL (*q* < 0.001).

**Figure 7:**
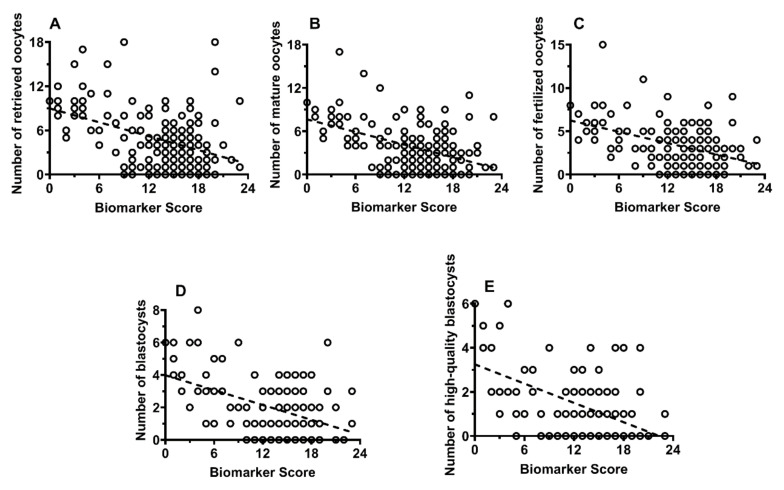
Linear correlations between the Biomarker Score values and number of retrieved oocytes (**A**), number of mature oocytes (**B**), number of fertilized oocytes (**C**), number of blastocysts (**D**), and number of high-quality blastocysts (**E**) in pooled FF donors, independently of initially being CTRL or IF. Spearman’s correlation coefficients were significant in any of the aforementioned associations (*p* < 0.001).

PCOS patients differed from CTRL only for the number of high-quality blastocysts (*q* < 0.05). The number of patients of all infertile subgroups, but PCOS and GI patients, resulting in positive to the β-HCG analysis were significantly lower than that of CTRL (*q* < 0.01). The Pearson’s correlation coefficients were calculated to evaluate whether the Biomarker Score influences the biological measures of IVF. To this purpose, all patients were combined into a single group of FF donors, since we assumed that the possible correlation between the Biomarker Score and the biological measures of IVF was independent of the patient’s clinical condition. As shown in Figure 7 (Panels A–E), significant values of the Spearman’s correlation coefficients were observed between the Biomarker Score and the number of retrieved oocytes (r = −0.373; *p* < 0.001), of mature oocytes (r = −0.375; *p* < 0.001), of fertilized oocytes (r = −0.363; *p* < 0.001), of blastocysts (r = −0.337; *p* < 0.001), and of high quality blastocysts (r = −0.346; *p* < 0.001), therefore, strongly indicating that biological parameters of IVF are inversely correlated with the Biomarker Score.

**Figure 8:**
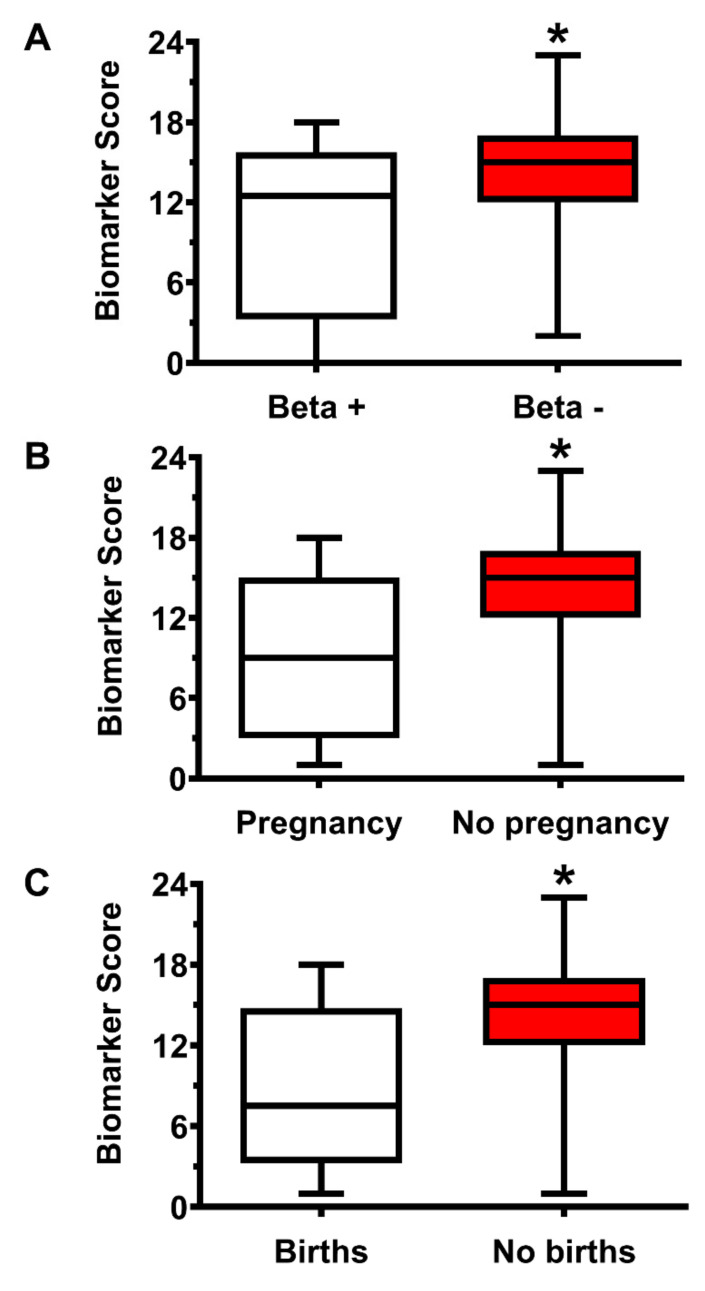
Box plots reporting the median, 1st and 3rd quartiles, minimum and maximum values of the Biomarker Score values in those FF donors who underwent embryo transfer and who were positive for β-HCG (**A**), who had a clinical pregnancy (**B**), and who delivered healthy offspring (**C**). FF donors were pooled into a single group, independently of initially being CTRL or IF. * Significantly different from beta +, pregnancy and births, *p* < 0.001.

Differences in the outcome measures of clinical pregnancy in CTRL and IF were clearly evidenced by indices related to fertilization and procreation capacities. In the group of CTRL, 25/25 blastocysts were transferred (100%). Following implantation, 15/25 patients (60%) were β-HCG positive, 11/25 developed clinical pregnancy (44%), and 11/25 generated healthy offspring (44%). In the group of IF 113/114 blastocysts were implanted (99.1%). In this case, only 26/114 patients (22.8%, *p* < 0.001 compared to CTRL) were β-HCG positive, 16/113 developed clinical pregnancies (14.2%; *p* < 0.001 compared to CTRL), and 11/109 generated healthy offspring (10.1%; *p* < 0.001 compared to CTRL). The influence of the Biomarker Score on outcome measures of effective IVF was assessed by considering patients with positive or negative β-HCG (scoring 1 or 0, respectively), those developing or not developing clinical pregnancies (scoring 1 or 0, respectively) and those who delivered or not delivered healthy offspring (scoring 1 or 0, respectively), regardless of the group they belong to (CTRL or IF). The Biomarker Score values of each patient were then paired to the corresponding outcome measures. Hence, comparisons were performed between the Biomarker Score values of patients with positive parameters (scoring 1) versus the Biomarker Score values of patients with negative parameters (scoring 0). The box plots in Figure 8 (Panels A–C) indicate that patients with positive indexes of outcome measures of effective IVF, independently of their clinical conditions, had lower values of the Biomarker Score, again suggesting that the biochemical pattern of metabolites, relevant for various cell functions, deeply affect the quality of the oocyte and the results of IVF, up to successful delivery of healthy offspring.

## 3. Discussion

By analyzing the FF to determine 55 water- and fat-soluble low molecular weight metabolites (using empowered Redox Energy Test), including energy metabolism substrates and products, purines, pyrimidines, hydrophilic antioxidants, oxidative/nitrosative stress biomarkers, lipophilic vitamins and antioxidants, and amino acids, the present study demonstrated significant anomalies in the FF composition of IF (clinically infertile because of EM, PCOS, AR-ROR, ROR, UI, and GI) compared to those found in CTRL. In particular, 27/55 compounds in FF of IF had values significantly different from those measured in CTRL, and these differences were also observed when IF were categorized according to the clinical diagnosis of infertility.

In the last decade, there was an increasing interest to evaluate the metabolic profile of FF in females affected by infertility in order to establish whether potential differences in the concentrations of specific compounds might be causative of, or related to infertility [21,22]. The final goal is to increase the efficiency of IVF since several clinical conditions leading to infertility (EM, PCOS, AR-ROR, ROR) are characterized by a low rate of success when these patients undergo IVF practice [23,24]. Furthermore, information on the biochemical composition of FF might be helpful not only for the comprehension of the molecular mechanisms of female infertility but also in setting up personalized therapies/diets aimed to modify the anomalous concentrations of metabolites in FF of IF [25,26].

The first notable result of the present study concerned glucose and lactate, the concentrations of which in CTRL were, respectively, 3.00 ± 0.45 mmol/L FF and 2.55 ± 0.60 mmol/L FF. Differently, we found that these compounds in pooled IF were, respectively, 2.15 ± 0.62 mmol/L FF (−28%, *p* < 0.001) and 3.87 ± 0.99 mmol/L FF (+52%, *p* < 0.001). With the exclusion of IF affected by PCOS and GI, the subgroups of those affected by EM, PCOS, AR-ROR, ROR, UI, and GI showed lower FF glucose values and higher FF lactate values than those measured in FF of CTRL (*q* < 0.005). There are no unanimous findings in previous studies on the concentrations of these energy-linked metabolites in FF of IF compared to those of CTRL. Results indicating either normal glucose and higher lactate [5], higher glucose and lower lactate [16], higher glucose and higher lactate [27], lower glucose and higher lactate [28,29,30], lower glucose and lower lactate [9], have been published in the last years. It is worth underlining that most of the aforementioned data have been carried out by selecting one specific category of female infertility.

In the present study, we first analyzed in blind FF from all donors, then calculated the concentrations of the various metabolites, and lastly matched the biochemical data with the clinical diagnosis of infertility (generating a group of CTRL and 6 subgroups of IF, according to the different clinical diagnoses). Our results indicate that lower glucose availability in FF occurs in pooled IF and in the IF subgroups of EM, AR-ROR, ROR, and UI, whilst PCOS and GI have normal glucose FF levels. Differently, an increase in FF lactate concentrations occurred in all IF subgroups, therefore appearing as a common biochemical feature of IF. These findings suggest that an imbalance between glucose consumption and glucose oxidation (presumably because of alterations of mitochondrial oxidative reactions), takes place during oocyte maturation in all IF, leading to an increase in the glycolytic rate with lactate overproduction. Some of the IF patients also showed a decrease in the efficiency of glucose transfer from the blood to FF. The energy deficit hypothesis seems to be corroborated by the 1.8 and 1.9 times increase in the concentrations, respectively, of hypoxanthine and xanthine in FF of IF (Table 2 and Figure S1, Panels C and D; *p* < 0.001 compared to CTRL), encountered in all subgroups after the clinical IF categorization (Figure 1, Panels C and D). This suggests an imbalance in ATP production and consumption with consequent activation of the adenine nucleotide degradation pathway and increased cellular efflux of free purine bases into the FF [31,32].

Numerous studies have previously found clear evidences of ROS and RNS overproduction in FF of IF with different clinical pathologies [33,34,35,36], although none of them performed the concomitant evaluation of the full profile of water-and fat-soluble antioxidants. In our group of IF, we found a significant decrease in FF concentrations of ascorbic acid, GSH, α-tocopherol, vitamin A, CoQ_10_, total carotenoids (*p* < 0.001), and a significant increase of MDA, 8-OH-dG, nitrite, and nitrate (*p* < 0.001). Interestingly, when categorizing the patients according to their clinical diagnosis, it was possible to observe common alterations of oxidative/nitrosative stress biomarkers but different patterns of antioxidant deficiencies, with EM and PCOS being more prone to show a decrease in fat-soluble antioxidants rather than in water-soluble antioxidants (Figure 1 and Figure 2).

Abnormalities in the concentrations of various AA in FF of females with different types of infertilities have been reported in several studies [37,38,39,40]. Although a previous research found an increase in several FF amino acids [41], our results clearly showed that IF are characterized by deficient concentrations of various amino acids in FF. Five of them (Ser, Thr, Val, Ile, and Leu) are indirectly involved, with their carbon skeleton, in cell energy metabolism [42,43] and one (Met) is directly involved in the so-called methyl cycle of fundamental relevance in many biochemical pathways [44,45,46]. It is conceivable to hypothesize that anomalous FF concentrations of these amino acids may affect not only oocyte development but, in the case of fertilization, even the embryo development, thus potentially resulting in an important cause of IVF failure.

The introduction of the Biomarker Score, obtained by using the 10–90% percentiles of the different metabolites measured in FF of CTRL reported in Table 2 and Table 3, allowed to cumulate into a single index the 27 metabolic/biochemical differences that characterize FF samples of IF from those of CTRL. According to this index, profitably used in other clinical contexts [19,31,47], CTRL has Biomarker Score values ranging from 0 to 9, whilst those of IF range from 8 to 23. Therefore, the Biomarker Score values permit to completely differentiate CTRL from IF and to infer that female fertility is associated with a maximum of 8/27 alterations of these metabolites in FF. The reliability of this index to discriminate subjects of the two groups of CTRL and IF are evidenced by the frequency of distribution of the Biomarker Score values, by the posterior probability curves of the Biomarker Score, and by the Biomarker Score ROC curve (Figure 4, Panels A, B, and C).

Further evidence of the relevance of the metabolic profile of the 27 compounds, discriminating the FF biochemical quality of CTRL from that of IF, was obtained when correlating the Biomarker Score values with biological parameters of oocyte and embryo developments. The strong negative correlations between the Biomarker Score and the number of retrieved oocytes, of mature oocytes, of fertilized oocytes, of blastocysts, and of high-quality blastocysts (Figure 7, Panels A–E), determined by pooling the data of all FF donors (independently on the group of CTRL or IF they belonged to), clearly indicate that the biochemical quality of FF strongly influences both the quantity and the quality of the oocytes, consequently affecting the subsequent possibility of correct embryo development. As aforementioned, since these correlations concerned all the subjects enrolled in this study, it appears evident that the FF levels of the 27 metabolites, and the consequent values of the Biomarker Score, are so much relevant for the oocyte quality to affect fertilization capacity even of CTRL.

Although the study produced very promising results, there are some limitations that should be clarified in the future. For instance, notwithstanding the number of pooled IF had a reasonable size (*n* = 145), when patients were categorized according to different clinical diagnoses of infertility, some of the six resulting subgroups included a small number of subjects. Therefore, it was not possible to discriminate the different subgroups of IF on the basis of their Biomarker Score values, even though it was possible to observe differences in specific FF metabolites characterizing some subgroups of IF (for instance, glucose in PCOS and ascorbate in EM and GI). As an additional study limitation, it should be considered that effecting the follicle pick-up when the follicle size is smaller than ≥18 mm (the follicle size of the pick-ups of our FF donors) different FF metabolic profiles might possibly be found. A further limitation is that the present results were obtained by measuring the various metabolites in pooled FF for those donors in whom more than one follicle was retrieved. This methodological choice, while being probably of minor relevance in differentiating CTRL from IF and from each of the six infertile subgroups, certainly limited the possibility to grade follicles from the same donor on the basis of the single follicle FF biochemical profile. Lastly, it should also be taken into account that the FF metabolic profiles might have been influenced by the different ovarian stimulation protocols, thus potentially acting as a confounding factor in the result interpretation.

In conclusion, according to the present results, it is possible to affirm that female infertility is characterized by anomalies in the concentrations of 27 compounds in FF, reflecting alterations in energy metabolism, antioxidant defenses, oxidative/nitrosative stress biomarkers, and free amino acids. These metabolic alterations are conveniently grouped into a single cumulative index, named the Biomarker Score, the values of which not only clustered CTRL (who, because of the couple infertility attributable to a male factor only, may be fairly considered as fertile FF donors) and IF into two distinct groups, but also correlated with biological outcome measures of oocyte and embryo development. Therefore, performing the metabolic profile of FF, and subsequently calculating the Biomarker Score value, would appear of great utility in the laboratory management of female infertility, also in light of potential personalized therapies aimed to selectively correcting the altered biochemical profiles of FF.

## 4. Materials and Methods

### 4.1. Patients’ Characteristics and Protocols for Ovarian Stimulation

The study was conducted according to the Declaration of Helsinki for Medical Research involving Human Subjects. Informed written consent was obtained from each FF donor enrolled in this study.

Patients included in the study were recruited at the Alma Res Fertility Centre (Rome, Italy) from September 2018 to January 2020, with the approval of the Alma Res Ethical Committee (approval number AREC0818FF). The groups of infertile females (*n* = 145) were unable to obtain pregnancy after at least one year of unprotected sexual intercourse. Control FF donors (CTRL, *n* = 35) were recruited among couples in which inability to obtain pregnancy was due to a male factor alone. Patients were included in the group of CTRL after a careful clinical assessment and only when the diagnosis of male infertility was clearly established. Patients were excluded from the study in the case of mechanical barrier in their reproductive system, previous history of cancer, and premature ovarian failure. Gynecological assessment, including hormone analysis, hysterosalpingography, and transvaginal ultrasound to evaluate the uterine cavity and antral follicle counts were used to categorize infertile females into the following groups: endometriosis (EM, *n* = 19); polycystic ovary syndrome (PCOS, *n* = 14); age-related reduced ovarian reserve (AR-ROR, *n* = 58); reduced ovarian reserve (ROR, *n* = 29); unexplained infertility (UI, *n* = 14); genetic infertility (GI, *n* = 11). In particular, patients were assigned to the PCOS subgroup according to the Rotterdam 2003 diagnostic criteria. The diagnosis of PCOS was confirmed when two of the following three criteria were included: (1) menstrual irregularities typical of ovulatory dysfunctions, mainly with oligomenorrhea or amenorrhea; (2) clinical hyperandrogenism, such as hirsutism, acne, alopecia, and/or biochemical hyperandrogenism, detected by laboratory hormone assays; (3) polycystic appearance of the ovary on ultrasound examination. Differently, patients were assigned to the EM subgroup when, on anamnesis, some of the following symptoms were noticed: dysmenorrhea, dyspareunia, chronic pelvic pain, menorrhagia, dysuria, and/or hematuria, and dyschezia and/or rectal bleeding. On physical examination, EM was evidenced by pain in the bimanual examination and fixed or not very mobile uterus (when this disease is profound and involves a massive formation of fibrotic processes). Laboratory investigations to support the diagnosis of EM included the dosage of the serum CA-125 marker, transvaginal ultrasound (to detect the presence of ovarian endometriomas, or endometriotic cysts of the ovary and adenomyomas, or endometriotic foci in the uterus wall), Nuclear Magnetic Resonance (to detect endometriotic foci in different locations of the pelvis). The combination of the results of these examinations/analyses strongly supported the diagnosis of EM, which was definitively performed after the histological examination of the biopsy of the endometriotic lesions. Furthermore, patients were assigned to the GI subgroup in the case of chromosomal aberrations, as well as in the case of mutations on genes known to cause infertility in females (BMP15, BMPR1B, CBX2, M33, CHD7, DIAPH2, FGF8, FGFR1, HFM1, FSHR, FSHB, FOXL2, FMR1, GNRH1, GNRHR, KAL1, KISS1R, GPR54, LHB, LHCGR, DAX1, NR5A1, SF1, POF1B, PROK2, PROKR2. RSPO1, SRY, SCNN1A, SOX9, STAG3, TAC3, TACR3, ZP1).

In CTRL, PCOS, UI, and GI, ovarian stimulation was induced by the administration of GnRH antagonist and menotropin starting on day 2 after the beginning of the cycle, followed by administration of human recombinant chorionic gonadotropin (r-HCG) (GnRH antagonist protocol). In EM, AR-ROR, and ROR, ovarian stimulation was induced by ultrashort flare GnRH analog starting on day 1 after the beginning of the cycle, followed by menotropin administration on day 2 after the beginning of the cycle, and by administration of r-HCG (ultrashort protocol). Patients underwent conventional in vitro fertilization (IVF) or intracytoplasmic sperm injection (ICSI), depending on the male factor and the type of infertility. Conventional IVF was applied in the case of unexplained infertility and endometriosis, whilst ICSI was performed in the case of earlier IVF failure or suboptimal fertilization, i.e., when the fertilization rate was lower than 20%, as well as in the case of male infertility with severe sperm abnormalities.

To reduce confounding factors, all participants (including controls) were interviewed to assess they had similar dietary patterns and lifestyles (all non-smokers, no one with alcohol or drug dependence, mild-to-moderate physical activity). Additionally, none of them was taking oral adjuvant/nutraceutical supplements during the 3 months before the analysis of their FF samples.

### 4.2. Collection of FF, Quantification of Ovarian Stimulation, Fertilization Procedures, Embryo Culture, Evaluation of Biochemical Pregnancy, Clinical Pregnancy, and Birth Rate

When at least three follicles reached an approximate size of ≥18 mm each, they were aspirated 36 h after the injection of r-HCG. Transvaginal, ultrasound-assisted follicles aspiration was performed with the vacuum pump. FF samples from the same donor were pooled, centrifuged for 10 min at 1500× *g* at room temperature and two aliquots of the resulting supernatants (500 μL each) were immediately processed for the analysis of metabolites, as subsequently indicated. Samples showing any visible trace of blood contamination were discarded.

Oocytes, denuded of surrounding cumulus cells by treatment with hyaluronidase (Vitrolife AB, Göteborg, Sweden), were inseminated via ICSI 2 h later, while oocytes via IVF were inseminated, without denudation, 4 h after oocyte retrieval. Injected oocytes were transferred to a culture dish containing 50 μL global total LP medium overlaid with 10 mL Ovoil (Vitrolife AB, Göteborg, Sweden) in a pre-equilibrated GPS-Embryo culture dish (CooperSurgical, Trumbull, CT, USA), and then cultured under an atmosphere containing 5% O_2_, 6% CO_2_, 89% N_2_ at 37 °C. At 5 days post-insemination, the number of developed blastocysts was counted and the quality of blastocysts was evaluated, in accordance with the Gardner blastocyst grading system. Blastocysts were then assessed for transfer or vitrification. 

For each patient, the number of retrieved oocytes, of mature oocytes, fertilized oocytes, blastocysts, high-quality blastocysts (defined as those scoring a grade ≥ 4AA), and positivity for β-HCG (1 = presence of β-HCG, 0 = no detectable β-HCG) were considered. In those patients who decided on the embryo transfer, the number of clinical pregnancies and of healthy offspring were also considered.

### 4.3. Samples Processing and HPLC Assays of Hydrophilic Low Molecular Weight Metabolites and Fat-Soluble Vitamins and Antioxidants

The targeted metabolomic evaluation of FF samples was carried out by the Redox Energy Test [19], empowered by the analysis of amino acids, glucose, lactate, and ammonia. To obtain protein-free FF extracts suitable for the HPLC determination of water-soluble metabolites, an aliquot of 500 μL of FF was supplemented with 1 mL of HPLC-grade acetonitrile, vortexed for 60 s and centrifuged at the maximum speed in a refrigerated top-bench centrifuge to precipitate proteins [48]. Supernatants were washed twice with large volumes of HPLC-grade chloroform to remove acetonitrile, centrifuged and the upper aqueous phases were transferred to different tubes, clearly labeled to identify the sample and stored at −80 °C until analyzed by HPLC to quantify the concentrations of different water-soluble compounds.

A second 500 μL FF aliquot was light-protected and then processed to extract fat-soluble antioxidants using a method recently described in detail elsewhere [49]. Briefly, samples were supplemented with 1 mL of HPLC-grade acetonitrile, vigorously vortexed for 60 s, and incubated at 37 °C for 1 h in a water bath under agitation to allow the full extraction of lipid-soluble compounds. Samples were then centrifuged at 20,690× *g* for 15 min at 4 °C to precipitate proteins and the clear supernatants were saved at −80 °C until the HPLC analysis of fat-soluble vitamins and antioxidants.

In all FF samples, the following water-soluble compounds related to purines and pyrimidines metabolism, energy metabolism, antioxidants, and oxidative/nitrosative stress were separated and quantified by HPLC according to methods set up in our laboratory [50,51]: cytosine, creatinine, uracil, β-pseudouridine, cytidine, hypoxhantine, guanine, xanthine, ascorbic acid, uridine, nitrite, reduced glutathione (GSH), inosine, uric acid, guanosine, malondialdehyde (MDA), nitrate, orotic acid, 8-hydroxy-2′-deoxyguanosine (8-OH-dG). Additionally, in all FF samples, the following free amino acids and amino group-containing compounds were separated and quantified by HPLC according to a pre-column ortophtalaldehyde derivatization methods set up in our laboratory [52]: aspartate (Asp), glutamate (Glu), asparagine (Asn), serine (Ser), glutamine (Gln), histidine (His), glycine (Gly), threonine (Thr), citrulline (Cit), arginine (Arg), alanine (Ala), carnosine (Car), taurine (Tau), γ-aminobutyrate (GABA), tyrosine (Tyr), S-adenosylhomocysteine (SAH), L-cystathionine (Cystat), valine (Val), methionine (Met), tryptophan (Trp), phenylalanine (Phe), isoleucine (Ile), leucine (Leu), ornithine (Orn), lysine (Lys).

In all organic solvent extracts of FF samples, the following fat-soluble vitamins and antioxidants were separated and quantified by HPLC [49,52]: *all trans*-retinoic acid, *all trans*-retinol (vitamin A), 25-OH-cholecalciferol (vitamin D_3_), α-tocopherol (vitamin E), γ-tocopherol, δ-tocopherol, coenzyme Q_10_, total carotenoids (calculated as the sum of astaxanthin + phytoene + lutein + zeaxanthin + *trans*-β-apo-8′-carotenal + β-cryptoxanthin + lycopene + α-carotene + β-carotene + violaxanthin).

The Surveyor HPLC apparatus (Thermo Fisher Scientific, Rodano, Milan, Italy) was equipped with a highly sensitive 5 cm light-path flow cell diode array detector and set up for acquisition between 200 and 550 nm wavelengths. Water-soluble compounds, free amino acids, and amino group-containing compounds were loaded (100 and 25 μL, respectively) onto a Hypersil C-18, 250 × 4.6 mm, 5 μm particle size column (Thermo Fisher Scientific, Rodano, Milan, Italy), while fat-soluble compounds (200 μL) were loaded onto a Hypersil Gold C-18, 200 mm × 4.6 mm, 5 μm particle size column (Thermo Fisher Scientific, Rodano, Milan, Italy). Both columns used were provided with their own guard columns. Data acquisition and analysis were performed using the ChromQuest^®^ software (version 5.0) package provided by the HPLC manufacturer.

### 4.4. Analysis of Glucose, Lactate and Ammonia in FF Samples

In all aqueous, protein-free FF extracts the concentration of glucose and ammonia were measured by automated procedures using the Cobas^®^ 6000 instrumentation (Roche Diagnostics, Basel, Switzerland). The amount of lactate in these FF extracts was determined spectrophotometrically using the method described by Artiss et al. [53,54].

### 4.5. Statistical Analysis

Statistical analysis was performed by using the GraphPad Prism program, release 8.0. Normality of distribution was tested by the Kolmogorov-Smirnov test. For all continuous variables, mean ± SD, median, minimum, maximum, quartiles (1st and 3rd), and percentiles (10% and 90%) were calculated, while frequencies were determined for categorical variables. Differences among the two groups of control subjects (CTRL) and infertile females (IF) were displayed by using the Mann-Whitney U-test and differences among CTRL and the groups of IF categorized into the 6 subgroups according to the clinical diagnosis (EM, PCOS, AR-ROR, ROR, UI, GI) were assessed by the 1-way ANOVA, followed by the two-stage linear step-up procedure of Benjamini, Krieger, and Yekutieli for multiple comparisons, as the post hoc test to control the false discovery rate. A *p* and *q* values of less than 0.05 were considered statistically significant.

For each of the metabolites showing significant differences between CTRL and IF (*n* = 27) and therefore being potentially considered as a biomarker for female infertility, we determined the 10% and 90% percentile of its concentration in each follicular fluid sample of the CTRL group. Subsequently, in the entire cohort of FF donors (CTRL and IF) each metabolite was assigned to one of the following two categories: (1) Normal (0) = when the metabolite concentration was ≥10% or ≤ 90% percentile of the CTRL group; (2) Positive (1) = when the metabolite concentration was outside the 10% or 90% percentile of the CTRL group. Subsequently, the concentrations of the various metabolites determined in the FF sample of each participant (of both CTRL and IF groups) were simplified into a specific profile of 0 (Normal) and 1 (Positive), lastly generating a “Biomarker Score” (sum of the number of Positive in each subject). The Biomarker Score, therefore, ranged from 0, when a subject scored 0 (Normal) for each of the metabolites identified as potential biomarkers, to 27 when a subject scored 1 (Positive) for each of the metabolites identified as potential biomarkers [19]. It is worth underlining that the 10–90% percentiles were chosen as the cut-off values for normality (instead of the more conventional 2.5–97.5% or 5–95% percentiles) because of the modest variability of the data found in CTRL. These cut-off values allowed to evidence reasonable numbers of 1 (Positive) subsequently used for the Biomarker Score calculation of all the FF donors, including those of the CTRL group.

In the preliminary analysis of the data, it was determined that the Biomarker Score values showed a normal distribution in both CTRL and IF groups. These normal distributions allowed calculation of the posterior probability curves using the Bayes theorem [20], thus permitting to outline whether a given subject belongs to the CTRL or IF groups, based on the individual value of Biomarker Score. Additionally, the ROC curve to show the specificity of the Biomarker Score value to assign a subject to the correct group (CTRL or IF) was also calculated.

To assess the correlation with the clinical parameters of fertility, subjects of the two groups were combined into a single group of FF donors and the value of the Biomarker Score of each subject was plotted as a function of the number of retrieved oocytes, of mature oocytes, of fertilized oocytes, of blastocysts, and of high-quality blastocysts. Linear correlations were calculated using Spearman’s correlation coefficient.

## Figures and Tables

**Table 1:** The clinical features and administration protocols carried out in the group of control subjects (CTRL) and in the six groups of infertile females categorized into endometriosis (EM), polycystic ovary syndrome (PCOS), age-related reduced ovarian reserve (AR-ROR), reduced ovarian reserve (ROR), unexplained infertility (UI,) and genetic infertility (GI).

Group of FF Donors	Age (Years)	BMI (kg/m^2^)	Serum FSH (IU/L)	Drug Administration Protocol	Number of Retrieved Oocytes	Number of Mature Oocytes
CTRL *n* = 35	30.6 ± 4.7	23.4 ± 5.1	7.9 ± 3.1	GnRH antagonist protocol	9.1 ± 3.5	7.4 ± 3.2
IF (pooled) *n* = 145	32.7 ± 7.6	24.6 ± 5.9	6.9 ± 3.0		3.9 ± 3.1 **	2.8 ± 2.5 **
EM *n* = 19	29.4 ± 5.5	24.2 ± 7.2	6.7 ± 2.4 *	Ultrashort protocol	4.7 ± 2.8 **	3.8 ± 2.4 **
PCOS *n* = 14	32.2 ± 6.8	24.8 ± 6.3	6.4 ± 3.2 *	GnRH antagonist protocol	8.4 ± 4.6	6.1 ± 2.8
AR-ROR *n* = 58	42.1 ± 5.3 *	27.6 ± 8.5 *	7.1 ± 2.8	Ultrashort protocol	3.1 ± 2.6 **	2.3 ± 2.2 **
ROR *n* = 29	28.8 ± 4.2	23.0 ± 3.9	6.7 ± 2.2	Ultrashort protocol	2.8 ± 2.4 **	1.7 ± 1.7 **
UI *n* = 14	35.1 ± 6.6	25.7 ± 5.8	8.2 ± 3.5	GnRH antagonist protocol	4.2 ± 2.8 **	2.5 ± 2.3 **
GI *n* = 11	28.5 ± 8.2	23.8 ± 4.4	7.4 ± 3.0	GnRH antagonist protocol	5.1 ± 3.3 **	3.6 ± 2.7 **

Values are the mean ± S.D. Ultrashort and GnRH protocols are detailed under Section 4. * Significantly different form corresponding values of CTRL (*q* < 0.01). ** Significantly different form corresponding values of CTRL (*q* < 0.001).

**Table 2:** Concentrations of water- and fat-soluble low-molecular-weight compounds representative of energy metabolism, purines, pyrimidines, antioxidant defenses, oxidative/nitrosative stress determined in deproteinized FF samples of control subjects.

Compound	Control Subjects (*n* = 35)	Reference Intervals (10–90% Percentiles)
NH_4_^+^	0.11 ± 0.05	0.06–0.16
Glucose	3.00 ± 0.45	2.40–3.60
Lactate	2.55 ± 0.60	1.70–3.30
Creatinine	72.98 ± 11.53	57.70–87.80
Hypoxanthine	0.57 ± 0.25	0.29–0.95
Xanthine	0.67 ± 0.31	0.30–1.18
β-pseudouridine	1.98 ± 0.70	1.04–2.84
Uracil	1.37 ± 0.56	0.73–2.30
Cytosine	0.21 ± 0.14	0.05–0.40
Cytidine	0.24 ± 0.13	0.07–0.41
Uridine	9.72 ± 2.85	5.00–12.50
Orotic acid	0.21 ± 0.27	0.00–0.73
Uric acid	287.38 ± 46.97	229.30–348.40
Inosine	0.58 ± 0.33	0.15–1.06
Adenosine	0.16 ± 0.23	0.00–0.34
Guanine	0.11 ± 0.12	0.00–0.27
Guanosine	0.22 ± 0.12	0.08–0.42
GSH	9.99 ± 4.41	5.20–17.50
Ascorbic acid	52.81 ± 15.57	36.25–72.70
MDA	0.029 ± 0.033	0.00–0.071
8-OH-dG	0.010 ± 0.010	0.00–0.020
Nitrite	2.07 ± 1.40	0.80–4.65
Nitrate	24.11 ± 9.30	15.30–33.20
*all-trans*-retinoic acid	0.012 ± 0.005	0.003–0.020
*all-trans*-retinol	4.89 ± 1.07	3.60–6.20
25-OH-cholecalciferol	0.066 ± 0.014	0.049–0.086
α-tocopherol	12.60 ± 2.18	9.70–16.10
γ-tocopherol	0.24 ± 0.08	0.14–0.36
Coenzyme Q_10_	0.046 ± 0.031	0.021–0.110
Total carotenoids	1.30 ± 0.46	0.78–2.00

Values of controls are the mean ± S.D. Concentrations of NH_4_^+^, glucose and lactate (including values of reference intervals) are expressed as mmol/L FF, whilst the remaining compounds (including values of reference intervals) are expressed as μmol/L FF. GSH = reduced glutathione; MDA = malondialdehyde; 8-OH-dG = 8-hydroxy-2′deoxy-guanosine; *all-trans*-retinol = Vitamin A; 25-OH-cholecalciferol = Vitamin D; α-tocopherol = Vitamin E; Total carotenoids = sum of astaxanthin + phytoene + lutein + zeaxanthin + *trans*-β-apo-8′-carotenal + β-cryptoxanthin + lycopene + α-carotene + β-carotene + violaxanthin. Sample processing, methods and HPLC conditions allowing the separation and quantification of the compounds under evaluation are described under Section 4.

**Table 3:** Concentrations of free amino acids determined by HPLC in deproteinized follicular fluid samples of control subjects.

Compound	Control Subjects (*n* = 35)	Reference Intervals (10–90% Percentiles)
Asp	37.78 ± 11.37	23.40–54.30
Glu	41.27 ± 11.63	28.10–56.90
Asn	34.07 ± 9.39	23.45–43.35
Ser	53.75 ± 19.39	38.35–74.80
Gln	407.41 ± 86.80	301.90–508.50
His	47.93 ± 10,63	35.40–62.00
Gly	258.10 ± 47.44	204.50–331.80
Thr	160.40 ± 37.10	120.20–227.20
Citr	15-06 ± 3.81	10.50–20.40
Arg	40.87 ± 6.86	30.80–48.90
Ala	226.10 ± 61.07	163.15–307.45
Carn	1.61 ± 1.46	0.40–3.85
Tau	19.40 ± 9.22	5.65–33.45
GABA	1.79 ± 1.24	0.60–3.90
Tyr	33.16 ± 8.77	20.75–44.25
SAH	4.59 ± 2.42	1.90–7.65
Cystat	11.20 ± 3.20	7.70–14.45
Val	192.70 ± 39.41	152.75–238.35
Met	21.73 ± 4.54	16.55–27.00
Trp	40.47 ± 5.80	32.25–49.30
Phe	46.65 ± 9.04	36.95–58.40
Ile	40.17 ± 7.31	31.90–50.15
Leu	68.72 ± 12.02	53.40–89.50
Orn	11.61 ± 4.31	6.80–18.55
Lys	86.30 ± 18.41	65.35–115.20

Values are the mean ± S.D. and, including values of reference intervals, are expressed as μmol/L FF. Asp = aspartate; Glu = glutamate; Asn = asparagine; Ser = serine; Gln = glutamine; His = histidine; Gly = glycine; Thr = threonine; Cit = citrulline; Arg = arginine; Ala = alanine; Carn = carnosine; Tau = taurine; GABA = γ–aminobutyrrate; Tyr = tyrosine; SAH = S-adenosylhomocysteine; Cystat = L-cystathionine; Val = valine; Met = methionine; Trp = tryptophan; Phe = phenylalanine; Ile = isoleucine; Leu = leucine; Orn = ornithine; Lys = lysine. Sample processing and HPLC conditions allowing the separation and quantification of the compounds under evaluation are described under Section 4.

**Table 4:** Values of the 27/55 compounds—the concentration of which in deproteinized follicular fluid samples of pooled IF were significantly different from that measured in the control subjects.

Compound	Control Subjects (*n* = 35)	Infertile Females (*n* = 145)
Glucose	3.00 ± 0.45	2.15 ± 0.62
Lactate	2.55 ± 0.60	3.87 ± 0.99
Hypoxanthine	0.57 ± 0.25	1.05 ± 0.74
Xanthine	0.67 ± 0.31	1.28 ± 0.74
β-pseudouridine	1.98 ± 0.70	3.06 ± 1.60
Uracil	1.37 ± 0.56	2.39 ± 1.60
Cytosine	0.21 ± 0.14	0.34–0.29
Cytidine	0.24 ± 0.13	0.44 ± 0.31
GSH	9.99 ± 4.41	6.42 ± 4.10
Ascorbic acid	52.81 ± 15.57	34.54 ± 20.16
MDA	0.029 ± 0.033	0.17 ± 0.12
8-OH-dG	0.010 ± 0.010	0.045 ± 0.055
Nitrite	2.07 ± 1.40	3.78 ± 3.34
Nitrate	24.11 ± 9.30	50.33 ± 25.10
*all-trans*-retinol	4.89 ± 1.07	3.84 ± 1.06
25-OH-cholecalciferol	0.066 ± 0.014	0.051 ± 0.021
α-tocopherol	12.60 ± 2.18	10.78 ± 2.86
Coenzyme Q_10_	0.046 ± 0.031	0.018 ± 0.017
Total carotenoids	1.30 ± 0.46	0.99 ± 0.43
Ser	53.75 ± 19.39	43.81 ± 15.40
Thr	160.40 ± 37.10	122.20 ± 37.48
Arg	40.87 ± 6.86	32.24 ± 9.34
Val	192.70 ± 39.41	161.40 ± 38.46
Met	21.73 ± 4.54	15.80 ± 5.67
Trp	40.47 ± 5.80	34.88 ± 7.41
Ile	40.17 ± 7.31	31.95 ± 9.47
Leu	68.72 ± 12.02	57.93 ± 13.93

Values are the mean ± S.D. Values in the column of control subjects are those already shown in Table 2 and Table 3. Concentrations of glucose and lactate are expressed as mmol/L FF, whilst the remaining compounds are expressed as μmol/L FF. GSH = reduced glutathione; MDA = malondialdehyde; 8-OH-dG = 8-hydroxy-2′deoxy-guanosine; *all-trans*-retinol = Vitamin A; 25-OH-cholecalciferol = Vitamin D; α-tocopherol = Vitamin E; Ser = serine; Thr = threonine; Arg = arginine; Val = valine; Met = methionine; Trp = tryptophan; Ile = isoleucine; Leu = leucine. Sample processing and HPLC conditions allowing the separation and quantification of the compounds under evaluation are described in Section 4. All values in infertile females were significantly different from those measured in control subjects, *p* < 0.001.

## Data Availability

Data available on request due to privacy and ethical restrictions.

## Supplementary Materials

The following are available online at https://www.mdpi.com/article/10.3390/ijms22168735/s1.
