# Supplementary material for: Altered Follicular Fluid Metabolic Pattern Correlates with Female Infertility and Outcome Measures of In Vitro Fertilization
Source: Int J Mol Sci. 2021 Aug 14;22(16):8735. doi: 10.3390/ijms22168735 (PMC8395780; doi:10.3390/ijms22168735)
Supplement: Supplementary file 1 [file ijms-22-08735-s001.zip › ijms-1273061-SI.pdf]

SUPPLEMENTARY MATERIALS

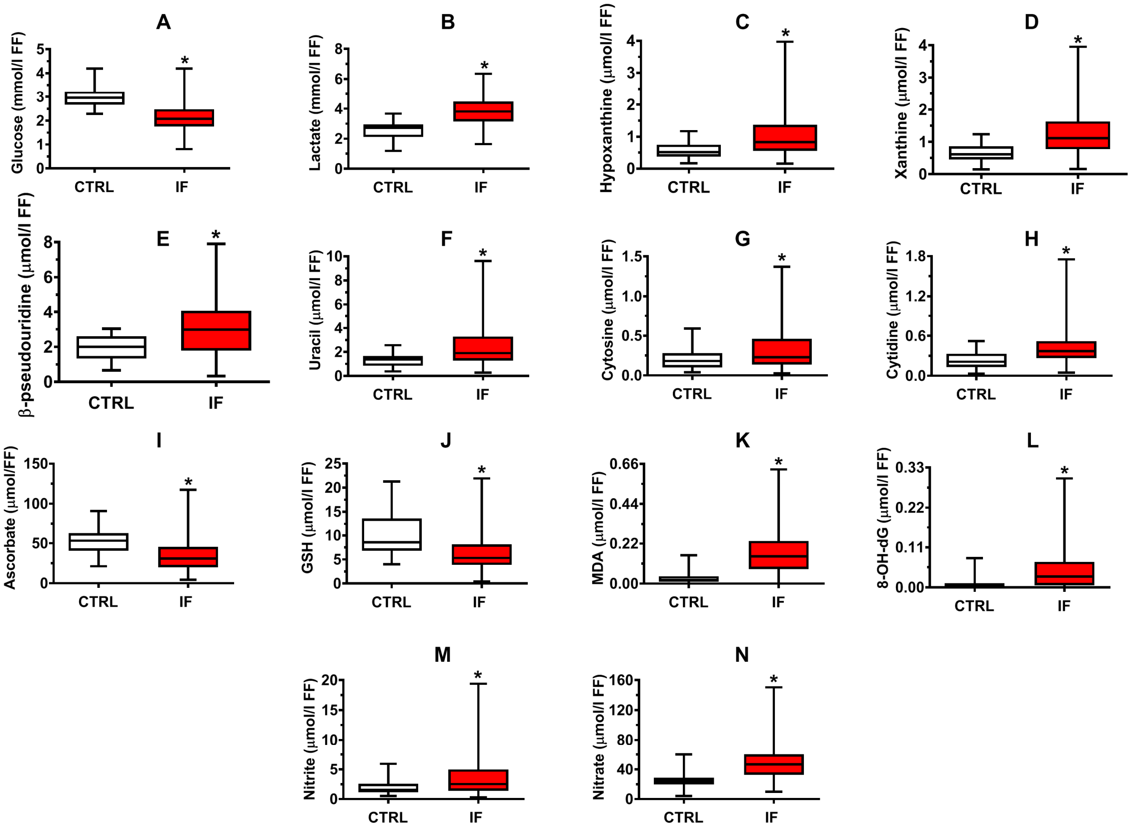

Figure S1. Box plots reporting median, 1st and 3rd quartiles, minimum and maximum values of the concentrations of glucose (A), lactate (B), hypoxanthine (C), xanthine (D), β-pseudouridine (E), uracil (F), cytosine (G), cytidine (H), ascorbate (I), GSH (J), MDA (K), 8-OH-dG (L), nitrite (M), and nitrate (N) detected in FF of 35 control subjectss (CTRL) and 145 infertile females (IF) pooled into a single group of infertility, independently on their clinical diagnosis. Energy related metabolites (A) and (B); purines ((C) and (D); pyrimidines (E)-(H); water-soluble antioxidants (I) and (J); oxidative stress indexes (K) and (L); nitrosative stress indexes (M) and (N).

*Significantly different from CTRL, $p < 0.01$.

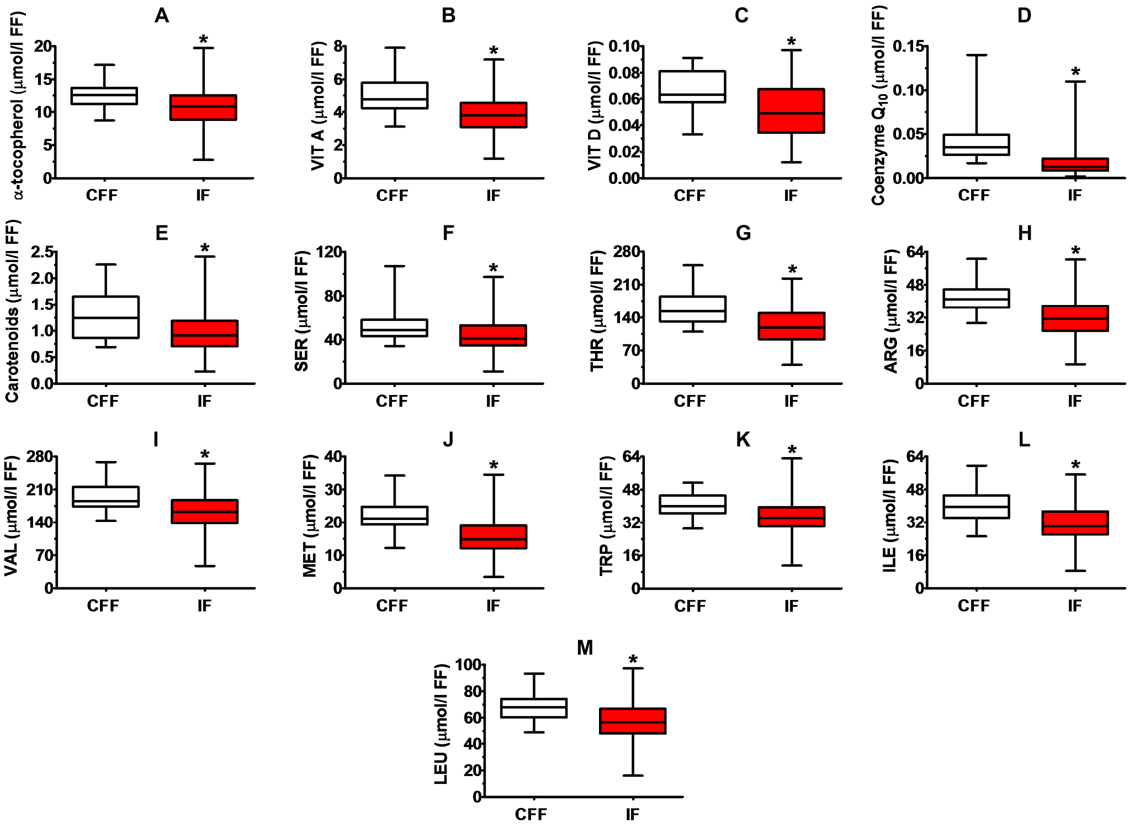

Figure S2. Box plots reporting median, 1st and 3rd quartiles, minimum and maximum values of the concentrations of α-tocopherol (A), vitamin A (B), vitamin D (C), coenzyme Q_{10} (D), carotenoids (E), serine (F), threonine (G), arginine (H), valine (I), methionine (J), tryptophan (K), isoleucine (L), and leucine (M) detected in FF of 35 control subjects (CTRL) and 145 infertile females (IF) pooled into a single group of infertility, independently on their clinical diagnosis. Fat-soluble vitamins and antioxidants (A)-(E); amino acids ((F)-(M).

*Significantly different from CTRL, $p < 0.01$.

Table S1. Raw data of the individual values of the Biomarker Score calculated in the 35 CFF and 145 pooled IF used to determine the ROC curve of Figure 4 (Panel C) of the main text.

CFF	IF
4	11
4	11
1	16
1	20
6	16
3	11
1	17
6	12
5	17
8	13
1	13
9	19
2	17
4	17
3	17
6	15
8	9
5	16
4	17
3	12
7	15

4	14
4	15
0	19
3	22
6	15
2	11
2	10
5	17
2	18
1	19
4	21
6	23
7	14
3	14
	18
	8
	14
	11
	11
	11
	15
	16
	13
	10
	12
	14
	20
	9
	10
	14
	14
	9
	16
	18
	13
	16
	10
	17
	8
	16
	18
	15
	15
	16
	11
	20
	15
	18
	11
	15
	18
	16
	17
	16
	15
	16
	16
	16
	10
	21
	13
	17
	16
	19
	15
	16
	14
	16
	17
	16
	13

12
9
16
16
13
13
16
18
12
15
14
16
16
17
14
15
10
11
15
17
16
12
13
15
15
17
15
22
15
10
14
14
12
20
19
15
16
17
12
19
22
13
14
13
15
14
11
15
14
17
11
17
15

Table S2. Parameters and statistic of the ROC curve (area under the curve, AUC) illustrated in Figure 4 (Panel C) of the main text, as calculated by GraphPad Prism 8.0 version.

Area under the ROC curve	
Area	0.9988
Std. Error	0.001154
95% confidence interval	0.9966 to 1.000
P value	<0.0001
Data	
Controls (CFF)	35
Patients (IF)	145
Missing Controls	0
Missing Patients	0

Table S3. Parameters used to calculate sensitivity and specificity of the ROC curve illustrated in Figure 4 (Panel C) of the main text, as calculated by GraphPad Prism 8.0 version.

	Sensitivity%	95% CI	Specificity%	95% CI	Likelihood ratio
> 5.500	100	7.49% to 100.0%	71.43	70% to 85.3	3.5
> 6.500	100	7.49% to 100.0%	85.71	74% to 95.1	7
> 7.500	100	7.49% to 100.0%	91.43	94% to 98.2	11.67
> 8.500	98.62	5.11% to 99.83%	97.14	08% to 99.9	34.52
> 9.500	95.86	1.21% to 98.47%	100	00% to 100.0%	
> 10.50	91.03	5.16% to 95.14%	100	00% to 100.0%	
> 11.50	82.76	5.61% to 88.52%	100	00% to 100.0%	
> 12.50	77.24	9.55% to 83.79%	100	00% to 100.0%	
> 13.50	69.66	1.48% to 77.01%	100	00% to 100.0%	
> 14.50	59.31	0.85% to 67.38%	100	00% to 100.0%	
> 15.50	44.14	5.91% to 52.61%	100	00% to 100.0%	
> 16.50	27.59	0.50% to 35.62%	100	00% to 100.0%	
> 17.50	15.86	0.33% to 22.84%	100	00% to 100.0%	
> 18.50	11.03	.440% to 17.30%	100	00% to 100.0%	
> 19.50	6.897	.356% to 12.32%	100	00% to 100.0%	
> 20.50	4.138	.533% to 8.789%	100	00% to 100.0%	
> 21.50	2.759	7566% to 6.912	100	00% to 100.0%	
> 22.50	0.6897	01746% to 3.782	100	00% to 100.0%	

Table S4. Parameters used to draw the ROC curve illustrated in Figure 4 (Panel C) of the main text, as calculated by GraphPad Prism 8.0 version.

100% - Specificity%	Sensitivity%
100.000	100.000
97.143	100.000
82.857	100.000
71.429	100.000
57.143	100.000
37.143	100.000
28.571	100.000
14.286	100.000
8.571	100.000
2.857	98.621
0.000	95.862
0.000	91.034
0.000	82.759
0.000	77.241
0.000	69.655
0.000	59.310
0.000	44.138
0.000	27.586
0.000	15.862
0.000	11.034
0.000	6.897
0.000	4.138
0.000	2.759
0.000	0.690
0.000	0.000
